# Enhanced High−Temperature Wear Performance of H13 Steel through TiC Incorporation by Laser Metal Deposition

**DOI:** 10.3390/ma16010099

**Published:** 2022-12-22

**Authors:** Chengqi Lu, Zhenyu Chen, Yuqing Yan, Yuhao Zhuo, Chuanyang Wang, Qingbo Jia

**Affiliations:** School of Mechanical and Electrical Engineering, Soochow University, Suzhou 215131, China

**Keywords:** laser metal deposition (LMD), H13 tool steel, composite coating, microstructure, high−temperature wear performance

## Abstract

High−temperature wear failure has been a major challenge to die parts. This work provides a comprehensive study on the high−temperature wear performance of a TiC/H13 composite coating prepared by laser metal deposition (LMD). The microstructures of wrought H13 samples, LMD−processed H13 and TiC/H13 samples were systematically investigated. The refined martensite size, the uniform distribution of TiC ceramic particles, as well as their bonding with the matrix endowed the fabricated composite coating with superior hardness. The LMD−prepared TiC/H13 composite coating material demonstrated outstanding wear resistance when compared with other counterparts, mainly due to the high thermal stability and the load-transferring effect triggered by the introduced TiC ceramic particles. The dominated wear mechanism transition from severe ploughing in the wrought H13 material to mild delamination in the TiC/H13 composite coating was confirmed. The present study is expected to shed light on high-temperature wear-resistant coating material design and applications within the highly demanding mould industry.

## 1. Introduction

H13 steel has been widely applied in the mould industry for the fabrication of extrusion dies, casting dies, and hot forming dies, mainly due to its excellent impact toughness, good thermal fatigue properties, and superior high-temperature strength [1,2,3]. However, serious mould surface issues have been observed after long-term usage at elevated temperatures (typically 500−600 °C), especially for the stress−concentrated edges and angular areas [4]. Take the hot stamping process as an example, the mould material generally experiences intense friction with the fabricated parts, making high-temperature wear failure a major challenge to die service. Therefore, the issues associated with the increasing demands for improving mould surface performance at elevated temperatures have been raised, with the final aim of prolonging the mould replacement cycle and reducing the corresponding economic losses.

To improve the die surface wear resistance at elevated temperatures, extensive efforts have been put to coat a hardened layer by means of chemical vapour deposition (CVD), physical vapour deposition (PVD) and laser surface treatment [5,6,7,8,9]. Among the various surface modification techniques, laser metal deposition (LMD) offers the advantages of modifying and/or remanufacturing the die surface with a significantly enhanced hardness, outstanding coating−substrate metallurgical bonding as well as a limited heat−affected zone [10]. The LMD process enables the precise control and versatile coating of a thin layer of materials onto the die surface with minimal distortion [11]. During LMD surface modification, the high−energy laser beam melts the coaxially injected metal powders and then rapidly solidifies them onto any local position of the part surface, including the edges and angular areas. Moreover, the rapid solidification within the generated molten pools endowed the deposited surface materials with fine microstructures and high hardness/wear resistance [12,13].

Ceramic particles−reinforced metal matrix composites (MMCs) provide multiple benefits of high strength−to−weight ratio, good wear resistance, and superior stiffness, which have received enormous attention in surface treatment industries [14,15,16]. The incorporation of ceramic particles, including WC, TiN, TiC, SiC, etc., onto iron-based alloys by laser surface treatment has been well documented [17,18,19,20]. The high thermal stability of ceramic particles also guarantees that the reinforced coating material has a good wear performance at elevated temperatures. Among all the introduced ceramic particles, TiC ceramic particles are regarded as the most preferred reinforcement due to their high stability, high hardness, and more importantly, good wettability with the steel matrix [21]. To date, many researchers have reported the enhanced surface properties of TiC−reinforced H13 tool steel made by laser surface treatment. Chen et al. investigated the laser-cladding TiC/H13 composites with various coarse TiC particle additions and found that the hardness can be significantly enhanced with an increase in TiC contents at a lower laser scanning speed [22]. Meng et al. showed that the hardness increased monotonically with the increase in TiC particles by bionic laser surface treatment, while its room−temperature wear resistance increased first and then decreased as a function of the TiC volume fraction [23]. Dadoo et al. revealed the process parameter effect on the shape and size of TiC and TiC−type MC carbides, and it was found that such carbides dominated the average hardness of the modified surface region [24]. Moreover, Jiang et al. demonstrated that the introduced TiC particles also promoted the enhancement of slurry erosion resistance at intermediate impact angles of 0−90° [25]. Though various benefits of TiC−reinforced H13 steel have been demonstrated by previous studies, its elevated temperature wear performance, especially at the commonly applied temperature range (500−600 °C) of H13 steel, still lacks systematic evaluation.

In this study, to evaluate the applicability of TiC ceramic particles for enhancing the high-temperature wear resistance of H13 steel, the TiC/H13 composite coating material was prepared by LMD. The role of the incorporated TiC particles on the microstructures and microhardness of H13 steel after the LMD process was systematically investigated. In comparison with the standard wrought H13 and the LMD−prepared H13 materials, the elevated temperature wear performance of the TiC/H13 composite material was studied, and the underlying wear mechanisms were elucidated in detail. The current work is expected to provide important scientific guidance for the application of LMD−prepared high−temperature wear-resistant coatings, and more importantly, shed light on future high−temperature wear-resistant coating material development for the highly demanding die industry.

## 2. Materials and Methods

### 2.1. Materials

**Figure 1:**
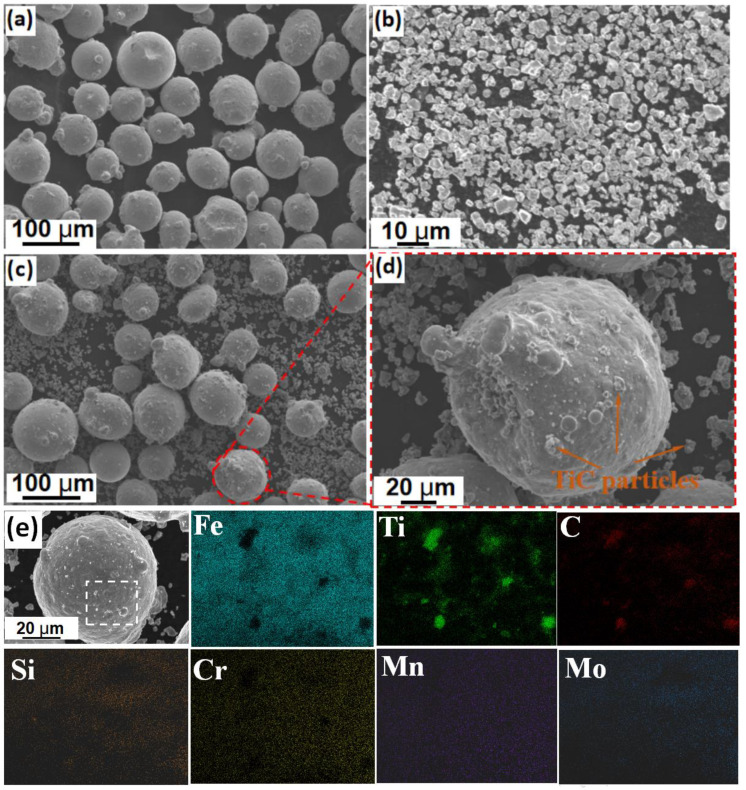
SEM images showing the morphology of (**a**) H13 powder, (**b**) TiC powder, (**c**) mixed powders, (**d**) magnified image of the attached TiC particles and (**e**) EDS mapping showing the element distribution in the attached TiC particles.

The applied H13 powder materials with a typical size range of 53–150 μm for the LMD process were prepared by argon gas atomization. To make the TiC/H13 composite powders, the TiC and H13 powders with a respective volume fraction of 10:90 were mixed in a planetary ball milling machine. As can be seen in Figure 1, the spherical H13 powder had a size distribution of 53–150 μm, while the granular TiC ceramic particles exhibited a typical size range of 3–5 μm. During the ball milling process, the powders were blended at a constant rotation speed of 350 r/min for total of 6 h, and the machine breaks every 20 min for about 10 min to guarantee the uniformity of the mixed powders. Moreover, it can be seen that some of the TiC particles were attached to the H13 powder surface after ball milling, which is expected to contribute to the uniform delivery of the composite materials to the formed molten pools (Figure 1d). For clarification purposes, the wrought H13 part is denoted as H13−W, while the laser metal deposited H13 and TiC/H13 are regarded as H13−L and TiC/H13−L, respectively. The chemical compositions of both the H13 powders and the wrought H13 bulk parts were verified by inductively coupled plasma atomic emission spectroscopy (ICP−AES), and the results are listed in Table 1. The bulk wrought H13 part, which was prepared in the water quench and tempered condition, was also studied for comparison purposes.

### 2.2. LMD Process

**Figure 2:**
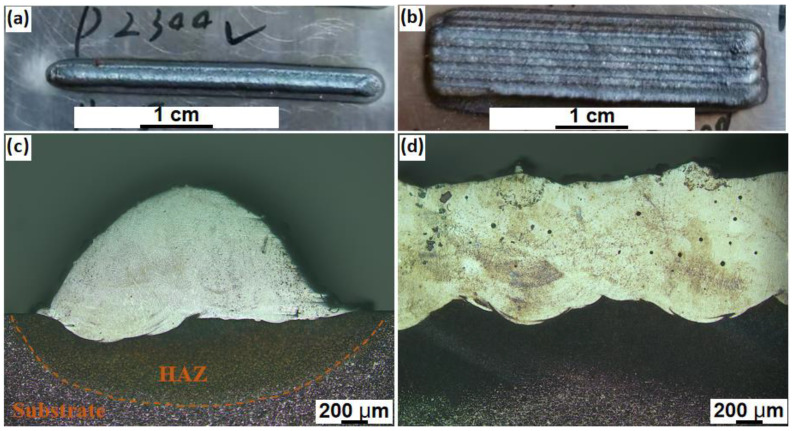
The representative (**a**) single−track and (**b**) multi−track morphology of the LMD−prepared H13 steel; (**c**,**d**) are the corresponding cross−section morphologies of (**a**,**b**).

The LMD process was performed with a 4 kW fibre laser system, where the powder feedstocks were delivered through an argon gas flow by a 5-axis robotic arm−controlled coaxial feeding nozzle. A piece of 45−carbon steel with a size of 100 mm × 100 mm × 10 mm was used as the substrate material, and the substrate surface was cleaned by ethanol before material deposition. To achieve the optimized process parameters, single−track experiments were first conducted to maintain a stable molten pool geometry and a good coating quality. The applied laser has a beam diameter of about 1.5 mm. After a series of process parameter trials, the optimized laser parameters were determined to be a laser power of 2300 W, a scanning speed of 13 mm/s, a disc rotating rate of 4 r/min for powder delivery and an overlap rate of 50%. In general, the optimized laser power density was calculated to be approximately 130 kW/cm^2^. Good surface finishes were obtained on both the single−track and multi−track samples (Figure 2a,b). The corresponding cross−sectional images confirmed the sound metallurgical bonding between the coated materials and the substrate, though occasional small gas pores with sizes below 100 um were observed (Figure 2c,d). Herein, the samples for cross−section morphology observations were etched in a solution composed of 10% nitric acid and 90% ethanol. The generated gas pores were mainly due to the entrapped protective argon gas within the molten pools under the open process environment.

### 2.3. Microstructural Characterization

Phase determination through X−ray diffraction (XRD) was conducted on a Bruker D8 Advance diffractometer with Cu−Kα (λ = 0.15046 nm) radiation, and operated at 40 kV and 40 mA with a scanning 2θ range of 20–100°and a step size of 0.02°. The samples for microstructural observations were all cut along the vertical sections of the deposited materials and then prepared with standard metallography procedures. A Zeiss Gemini 300 scanning electron microscope (SEM) equipped with a Bruker EDS setup was applied for grain structure and carbide investigations. Electron backscattered diffraction (EBSD) images of the sample cross−sectional areas were revealed by an OXFORD Symmetry S detector installed in the SEM equipment. Moreover, the worn track surface areas of the sample after high−temperature wear tests were also analyzed using SEM equipment. The worn track surface was further reconstructed using a Keyence VHX−7000 3D optical profilometer.

### 2.4. High−Temperature Abrasive Wear Test

The microhardness values along the sample depth direction were recorded on a Vickers 1000EG hardness tester under a loading force of 100 g and a loading time of 15 s. The high−temperature wear behaviours of the studied materials were evaluated on a Bruker UMT−TL REC−1000 °C tribometer in a reciprocal manner. The corresponding wear counterpart was made of Si_3_N_4_ ceramic balls, and the friction contact form was then determined as ball−on−disk. Three sets of wear experiments were conducted for each polished sample to ensure the repeatability of the tested data. The high−temperature wear tests were all conducted at 600 °C with a normal load of 50 N and a sliding speed of 5 mm/s, and the total testing time was 15 min. Before wear testing, the samples and the wear counterparts were first heated to the target temperature and then maintained at this temperature for at least 10 min to ensure the whole sample reached the target temperature. The coefficient of friction (COF) was automatically recorded from the tribometer during testing. The wear depth and width were determined based on the analysis of the 3D worn-surface track morphology, while the wear volume was then calculated by integrating the cross−sectional areas of the worn tracks.

## 3. Results and Discussion

### 3.1. Phase Identification

**Figure 3:**
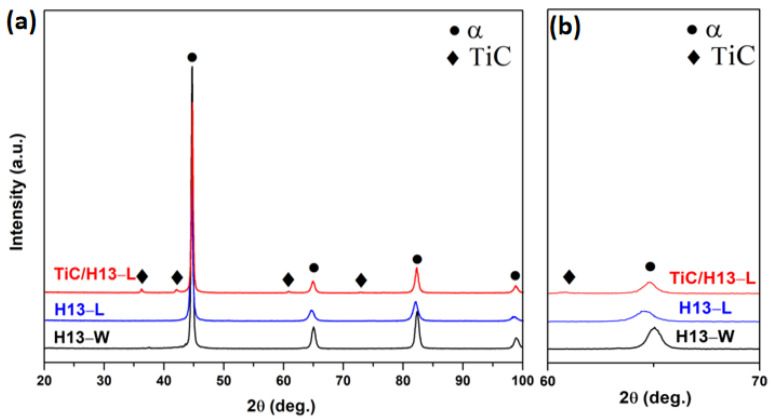
(**a**) XRD profiles showing the diffraction peaks of the captured phases in the studied materials, (**b**) is an enlarged view of (**a**) at the scanning 2θ angle of 60−70°.

The XRD analyses were performed to reveal the presented phases in the prepared samples (Figure 3). It can be seen that the major diffraction peaks corresponding to the α−martensite phase were found in all the studied samples. The incorporated TiC particles were also successfully captured in the LMD−fabricated TiC/H13 composite coating. The enlarged view across the 2θ scanning range of 60–70° is shown in Figure 3b. Obviously, the α phase peak became broadened for the LMD−prepared samples compared to the wrought counterpart, indicating the significantly refined microstructures after the laser process [26]. Additionally, peak shift was also observed for the LMD−prepared samples, indicating expanded lattice planes. This can be attributed to either the enhanced solid solution effect or the residual strain effect. To verify this, based on the William−Hall method [27], the calculated residual microstrains were 0.00277, 0.00316 and 0.00173 for the H13−W, H13−L and TiC/H13−L samples, respectively. It is worth noting that the peak shift amplitude of the TiC/H13−L sample was slightly lower than that of the H13−L sample. Previous studies have demonstrated that the incorporation of ceramic particles can markedly improve the laser absorbing efficiency, but lowers the overall thermal diffusion effect of the melted material [28]. Thus, the cooling rate and the resultant residual stress/solid solution effect of the TiC/H13-L sample might be lower than that of the H13−L, causing the above peak shift variances.

### 3.2. Microstructures

**Figure 4:**
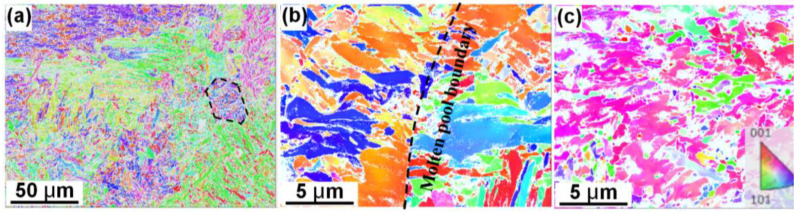
Inverse pole figure maps showing the martensite microstructures of (**a**) the H13−W, (**b**) H13−L and (**c**) TiC/H13−L samples.

Figure 4 shows the EBSD analyses of the inverse pole figures of the studied samples along the cross−sectioned areas. The fine martensite structure within the retained austenite grain boundary regions (as represented by the dashed lines) was captured in the H13−W sample (Figure 4a). On the other hand, the austenite grain boundary regions can be hardly defined in the LMD−processed H13 sample due to the turbulence of melt flow and the resultant non−equilibrium solidification. Moreover, the martensite microstructure also exhibited an epitaxial growth along the molten pool boundary (Figure 4b). Herein, it is worth noting that the retained austenite was not identified by XRD patterns mainly due to their limited volume fraction. This could have been a result of the high−temperature gradient and/or the large undercooling within the laser-induced molten pools, which greatly restricted the growth of the austenite. The incorporation of the TiC particles, on the other hand, further refined the martensite microstructures. This is because either the introduced TiC particles worked as heterogeneous nucleation sites or inhibited the rapid growth of the austenite structures (Figure 4c). Herein, the majority of the unindexed area of the TiC/H13−L sample was mainly due to the existence of small TiC particles.

**Figure 5:**
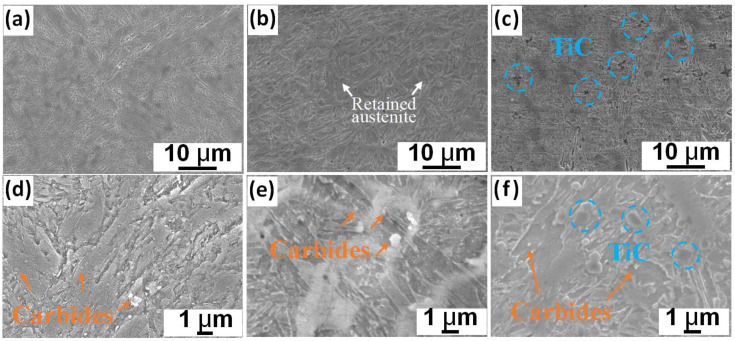
SEM images showing the microstructures composed of retained austenite, martensite and carbide particles in (**a**,**d**) H13−W, (**b**,**e**) H13−L and (**c**,**f**) TiC/H13−L samples.

SEM images further demonstrated the above gradual refinement trend of the martensite microstructures. The typically tempered martensite was observed in the H13−W sample, and higher−magnification images revealed the granular carbides and the retained austenite along the martensite structure boundaries (Figure 5a,d). In fact, the studied H13 steel normally contains various carbides (including the M_23_C6, VC, and Mo_2_C) with sizes measuring from dozens to hundreds of nanometers, and such particles can be regarded as main strengthening contributors at elevated temperatures [29,30]. On the other hand, the fine martensite structures distributed across the retained austenite matrix in the LMD−processed H13 sample were identified. Compared with the H13−W sample, the relatively larger area of the retained austenite indicates insufficient time for austenite to martensite phase transformation due to the rapid cooling rate of the laser process. Moreover, lath martensite with granular carbides was found after closer investigation. The amount of retained austenite, however, can still be further lowered with the addition of the TiC particles under the LMD process. As stated above, this is mainly because the introduced TiC ceramic particles reduce the thermal diffusion effect that promotes the solid-state phase transformation during the cooling process. It is worth noting that the introduced TiC particles generally exhibited a relatively uniform distribution behaviour within the alloy matrix. Apart from their homogeneous distribution in the initial powder feedstocks, previous studies also showed that the Marangoni convection and the associated thermal fluid flow can also promote the rearrangement and distribution behaviours of the ceramic particles [31]. Interestingly, the TiC particles are slightly refined after the LMD process compared to the initial powders. We suggest that this can be a result of the partial melting of the particle edges within the extremely high−temperature molten pools. This can be further supported by the good bonding between the TiC particles and the alloy matrix (Figure 5f).

### 3.3. Microhardness

**Figure 6:**
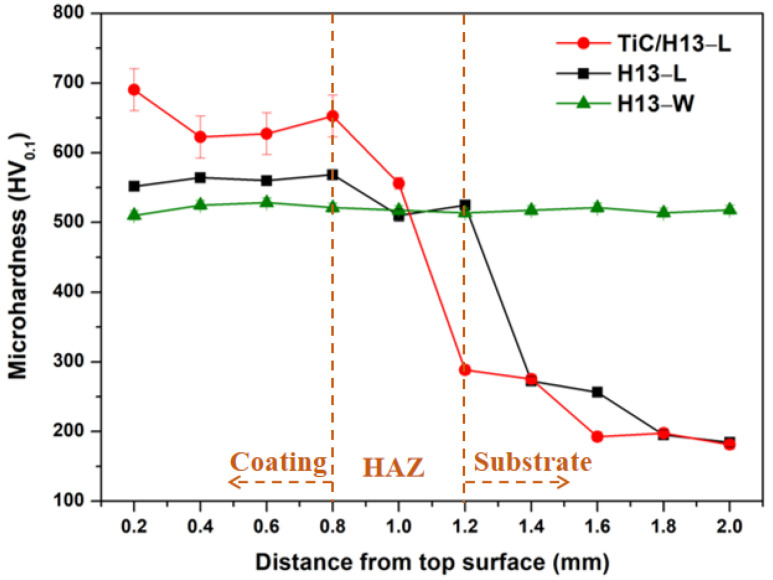
Microhardness distribution along the cross−section of the studied samples.

The microhardness value distributions along the cross-sections of the LMD−processed alloys are shown in Figure 6, and the data for wrought H13 alloy is also included for comparison purposes. In general, the hardness value showed a continuous decreasing trend from the coating area to the heat-affected zone (HAZ) and finally the substrate (45−carbon steel) area. Moreover, when compared with the wrought H13 alloy, the average hardness value of the LMD-prepared coatings increased by about 30% and 10% for the TiC/H13−L and H13−L samples, respectively. Furthermore, from the hardness changing behaviour of the HAZ, it can be found that the depth of the HAZ in TiC/H13−L is relatively low compared to H13−L, possibly due to the thermal diffusion variance induced by the introduced ceramic particles. This will be clarified in future studies.

Clearly, the markedly refined martensite lath resulting from the high cooling rate of the laser process contributed to the hardness improvements in the LMD-fabricated coatings to a great extent. On the other hand, the in situ−formed carbide particles with sizes below a few hundred nanometers of H13 steel would have been expected to provide a strong strengthening effect by inhibiting dislocation motion. Moreover, due to the good bonding behaviour between the TiC particles and the alloy matrix, the load−transferring effect offered by the ceramic particles further elevated the global strength of the composite coating material [32].

### 3.4. High−Temperature Wear Performance

**Figure 7:**
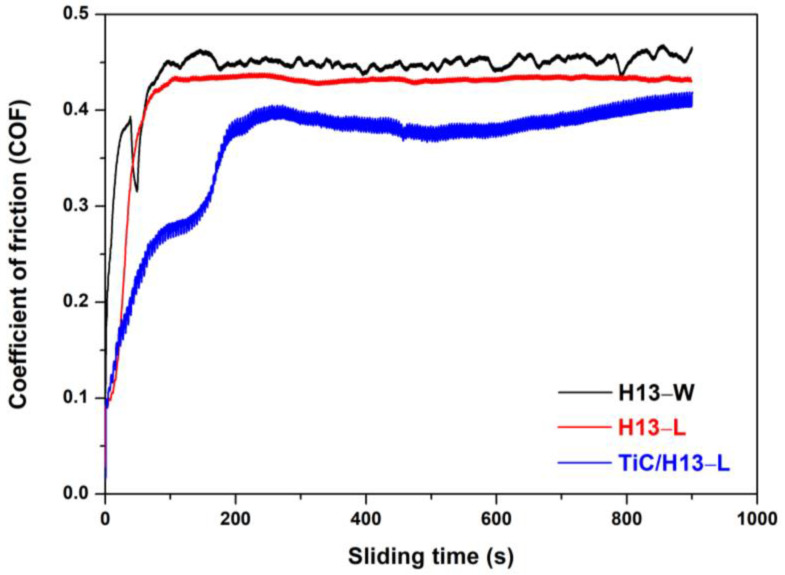
The COF values of the studied samples during elevated temperature wear testing at 600 °C.

The LMD−fabricated coating materials and the wrought H13 alloy were subjected to wear testing at an elevated temperature of 600 °C. Figure 7 shows the COF curves for the studied materials. Overall, the COF values exhibited first a rapidly increasing trend and then maintained a stable value. More specifically, the COF of the H13−W and H13−L samples maintained respective stable values of 0.45 and 0.43, respectively, after sliding for about 100 s. On the other hand, the COF of the TiC/H13−L sample demonstrated a much slower initial increasing trend, in which a small transition plateau at a sliding time of 100 s was observed. Afterwards, the sample experienced further increases, but overall maintained a COF value below 0.4. Herein, it can be deduced that a stable surface condition might be destroyed in the composite coating material, suggesting a possible wear mechanism transition during the initial wear testing.

**Figure 8:**
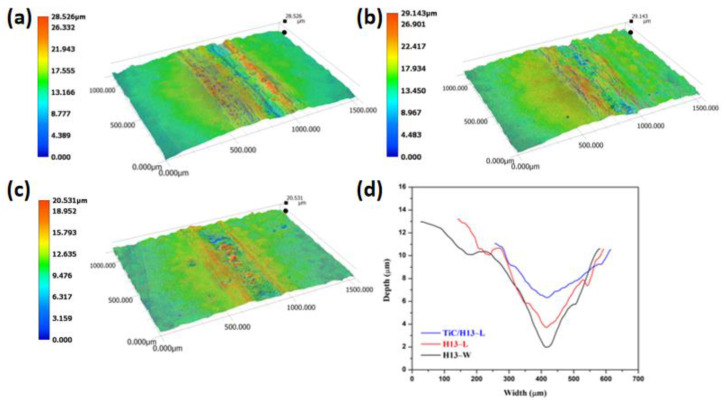
The 3D worn track morphologies of (**a**) H13−W, (**b**) H13−L and (**c**) TiC/H13−L samples, and (**d**) the extracted wear track width/depth profiles of the above-tested samples.

**Figure 9:**
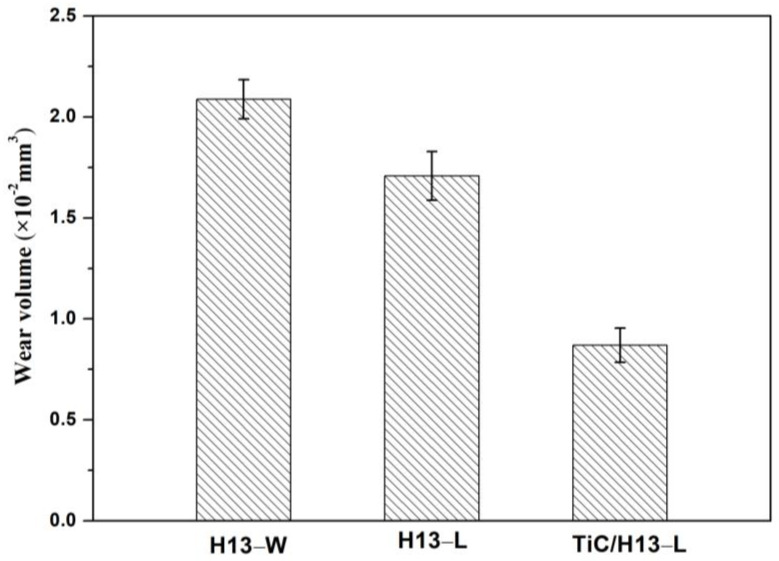
The calculated wear volume from the 3D wear track profiles of the studied samples.

The worn tracks of the above−studied materials were further analysed using 3D optical profilometry. The representative 3D wear track morphology and wear track width/depth profiles are shown in Figure 8. It can be seen that the H13−W sample has a rugged worn area with deep grooves in the middle area of the wear track. Though the wear track of the LMD−prepared H13 sample is still uneven, it showed a wear mechanism transition and a wear performance enhancement with the absence of the deep grooves. By further incorporating the micron-sized TiC particles into the coating material, a uniform and shallower wear track was observed. The measured wear track profile confirmed that the wear H13−W sample had a maximum worn track depth of approximately 11 um and a width of 556 um, in comparison to the TiC/H13 sample which had a maximum worn track depth of approximately 4.8 um and a width of 360 um. Moreover, based on the wear track profiles, the corresponding wear volumes of the H13−W, H13−L and TiC/H13−L samples were calculated to be 2.08 × 10^−2^ mm^3^, 1.7 × 10^−2^ mm^3^ and 0.87 × 10^−2^ mm^3^, respectively (Figure 9).

**Figure 10:**
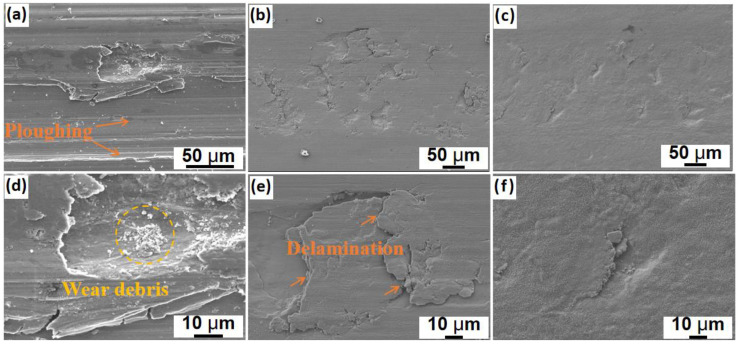
The worn-surface morphologies of (**a**,**d**) H13−W, (**b**,**e**) H13−L and (**c**,**f**) TiC/H13−L samples after high−temperature wear testing at 600 °C.

To further illustrate the wear mechanisms, the wear track morphologies were characterized by SEM (Figure 10). The wrought H13 sample was characterized by severe ploughing across the whole wear track, indicating insufficient material strength at elevated temperatures. Moreover, intense material delamination and removal from the wear tracks were frequently observed, and higher−magnification images revealed the wear debris within the damaged surface areas. Thus, the dominant wear mechanisms for the H13−W sample can be ascribed to ploughing at the applied testing temperature. On the other hand, when compared with the wrought H13 sample, the H13−L sample showed the absence of grooves on the wear surface. Instead, the wear mechanisms become delamination dominated, though the material loss is still serious. The incorporation of TiC particles into the H13 matrix significantly improved the worn-surface quality, and the majority of the wear surface maintained its integrity under the above same wear testing conditions. In fact, only several micropits were observed on the wear surface of the TiC/H13−L composite coating. Higher−magnification images revealed that the material delamination was successfully suppressed and no obvious wear debris was captured.

The above study demonstrated the effective wear resistance enhancement of LMD−prepared TiC/H13 composite coatings. Without a doubt, the markedly improved microhardness due to the uniform distribution of TiC ceramic particles contributed to the wear resistance enhancement when confronting its ceramic wear counterparts. Though the hardness can also be improved by the refined martensite in the H13−L sample, the wear loss reduction is rather limited due to material softening at elevated temperatures. The added TiC particles, on the other hand, acted as strong strengthening contributors due to their high thermal stability at elevated temperatures. It is also proposed that the thermally stable TiC particles can lower the real contact area between the wear pairs, elevating the wear resistance of the matrix material [33]. Moreover, the good bonding behaviour guarantees the activation of the load−transferring effect between the hard TiC particles and the relatively soft H13 steel matrix, which prohibited material delamination and removal from locally worn areas. Furthermore, the introduction of TiC particles can also promote the oxidation resistance of the composite coating, as the oxide layers were found to be worn out easily during elevated temperature wear testing [34]. In all, we suggest that LMD−prepared TiC/H13 composite coatings can be promising routes in the fabrication of high-temperature wear-resistant coatings.

## 4. Conclusions

In this study, the high-temperature wear behaviours and wear mechanisms of LMD−prepared TiC/H13 coatings were comprehensively studied. The following conclusions can be drawn:

(1) The LMD-prepared TiC/H13 composite coating possessed refined martensite microstructures compared to the H13−W and H13−L samples, which could be a result of the high-temperature gradient, high undercooling, as well as the introduced TiC particles. The TiC ceramic particles demonstrated a finer size, uniform distribution, and good bonding with the H13 steel matrix.
(2) The TiC/H13 composite coating exhibited a superior microhardness and high-temperature wear performance than its LMD- and wrought−processed H13 counterparts. The microhardness value for the composite TiC/H13 coating increased by nearly 30% more than the H13-W sample, e.g., from 508 HV_0.1_ to 648 HV_0.1_, while its wear track depth/width (from 11 μm/556 μm to 4.8 μm/360 μm) and wear volume (from 2.08 × 10^−2^ mm^3^ to 0.87 × 10^−2^ mm^3^) decreased significantly compared to its wrought counterpart.
(3) Compared with the wrought H13 sample, the high microhardness and the high thermal stability of the LMD−prepared TiC/H13 composite coating promoted the dominant wear mechanism transformation from ploughing in the H13−W sample to mild delamination in the TiC/H13−L sample.

Overall, this study demonstrated the effective high-temperature wear performance enhancement of LMD−prepared TiC/H13 composite coatings, and we expect the methodology applied in this study can provide some useful guidance in future wear−resistant die surface repair or remanufacture.

## Figures and Tables

**Table 1:** The chemical compositions of the wrought H13 sample and the H13 powders.

Elements (wt%)	Cr	Mo	Mn	C	Si	V	P	S
Wrought H13	4.98	1.35	0.39	0.36	0.86	0.78	0.021	0.003
H13 powder	5.44	1.29	0.38	0.34	0.92	1.03	0.008	0.022

## Data Availability

The data presented in this study are all available in the paper.
